# Association between Self-Reported and Accelerometer-Based Estimates of Physical Activity in Portuguese Older Adults

**DOI:** 10.3390/s21072258

**Published:** 2021-03-24

**Authors:** Célia Domingos, Nadine Correia Santos, José Miguel Pêgo

**Affiliations:** 1 Life and Health Sciences Research Institute (ICVS), School of Medicine, University of Minho, 4710-057 Braga, Portugal; id6512@alunos.uminho.pt (C.D.); nsantos@med.uminho.pt (N.C.S.); 2 ICVS/3B’s, PT Government Associate Laboratory, 4710-057 Braga/Guimarães, Portugal; 3 iCognitus4ALL-IT Solutions, 4710-057 Braga, Portugal; 4 Clinical Academic Center-Braga (2CA-B), 4710-057 Braga, Portugal; 5 ACMP5-Associação Centro de Medicina P5 (P5), School of Medicine, University of Minho, 4710-057 Braga, Portugal

**Keywords:** seniors, elderly, physical activity, accelerometry, fitness trackers, self-report physical activity, IPAQ, YPAS

## Abstract

Accurate assessment of physical activity (PA) is crucial in interventions promoting it and in studies exploring its association with health status. Currently, there is a wide range of assessment tools available, including subjective and objective measures. This study compared accelerometer-based estimates of PA with self-report PA data in older adults. Additionally, the associations between PA and health outcomes and PA profiles were analyzed. Participants (*n* = 110) wore a Xiaomi Mi Band 2^®^ for fifteen consecutive days. Self-reported PA was assessed using the International Physical Activity Questionnaire (IPAQ) and the Yale Physical Activity Survey (YPAS). The Spearman correlation coefficient was used to compare self-reported and accelerometer-measured PA and associations between PA and health. Bland–Altman plots were performed to assess the agreement between methods. Results highlight a large variation between self-reported and Xiaomi Mi Band 2^®^ estimates, with poor general agreement. The highest difference was found for sedentary time. Low positive correlations were observed for IPAQ estimates (sedentary, vigorous, and total PA) and moderate for YPAS vigorous estimates. Finally, self-reported and objectively measured PA associated differently with health outcomes. Summarily, although accelerometry has the advantage of being an accurate method, self-report questionnaires could provide valuable information about the context of the activity.

## 1. Introduction

Regular physical activity (PA) is essential for healthy aging and chronic disease prevention, with its benefits on general health and overall quality of life in older adults being well established [1,2]. Specifically, PA contributes to maintaining physical function and performance (e.g., walking speed, handgrip strength) [3] and plays a preventing role in frailty and falls [4,5]. Furthermore, it associates with more favorable body composition and anthropometric parameters such as body mass index (BMI), waist circumference, and weight [6]. There is also increasing evidence of its beneficial effects on various neuropsychological outputs [7]. Despite this, the quantification of the strength and nature of the relationship between PA and health outcomes should rely on the accurate measurement of PA behavior [8].

In population-based studies, there are currently several objective and subjective self-report instruments available [9], but with accumulating scientific evidence being mainly based on the latter (e.g., self-report questionnaires, logs, recalls, and activity diaries) [1,10]. Nonetheless, recalling PA is a complex cognitive task, particularly for older adults who may have memory and recall limitations [1]. Indeed, self-report questionnaires have limited validity in measuring daily activities due to issues with question interpretation, imprecise recall, judgment formation, and response editing [8,11,12]. On the other hand, self-report measures are more feasible for use in large-scale studies because of their low cost and ease of use [8,9,12,13,14]. Moreover, questionnaires can provide information on the type (e.g., leisure, household, work, transportation) and duration of PA (e.g., min.day^−1^, min.wk^−1^, h.wk^−1^) [12]. Still, and complementing this type of methodology, more objective PA measurement is of concern. Across time and populations, accelerometers are reliable and valid objective instruments [1,11,15], providing a precise assessment of everyday activities (frequency, duration, intensity, and type), and allowing for the identification of specific PA patterns [11,15,16], without the need for self-report [11]. However, they are unable to capture all PA dimensions, such as leisure, household, work, and transportation activities [12].

Previous studies using both measurement types have observed some differences in the obtained PA estimates [9]. In general, self-report measures both underestimate sedentary time [17] and overestimate PA levels when compared to PA objective measures [17,18,19,20]. Furthermore, research conducted on the general population has suggested self-reported measures of PA as inaccurate and showing a weak to modest correspondence when compared with objective measures [13,14,21]. Therefore, one of the challenges is to select the most accurate and practical tool with a balance between precision and feasibility [8], both in research and in clinics. Notably, poor methods increase the chances of misclassification and can mask or distort the true underlying relationship between PA and health outcomes [8]. This can be particularly notable in what regards accelerometer and self-report measures in sedentary behavior [17]. Additionally, there is a lack of knowledge about the PA levels and sedentary behavior among the elderly [1] and, despite all the evidence supporting their benefits in health outcomes, there is a need to clarify the strength of this relationship. Thus, comparison studies between accelerometry and self-report methods are essential.

In a sample of community-dwelling of Portuguese older adults in Northern Portugal, here the study objectives were to (i) compare PA assessed by commonly used self-report questionnaires versus objective PA assessed by accelerometry, and (ii) describe the levels of PA and explore their associations with body composition and physical function. Results may provide further insight on accelerometer measurements and PA levels and sedentary behavior in the senior population, contributing to the future implementation of this wearable technology in clinical and research settings.

## 2. Materials and Methods

### 2.1. Ethics Statement

The study was approved by the local ethical committees (Approval Number 42-2018), conducted in accordance with the Declaration of Helsinki (59th Amendment), developed in compliance with the new General Data Protection Regulation, and approved by the Portuguese Data Protection Authority (Approval Number 11286/2017). The study goals and assessments were explained to participants during screening procedures. Written informed consent was obtained from all participants prior to the study enrollment.

### 2.2. Participants

A convenience sample of 120 community-dwelling older adults were recruited from local health centers and gyms from the municipality of Braga in Northern Portugal. The eligible criteria were: (1) men and women between 64 to 75 years of age; (2) ability to understand the informed consent; (3) ability to attend the assessment sessions; (4) ability to wear Xiaomi Mi Band 2^®^ for fifteen consecutive days; (5) free of diagnosed neurological or psychiatric diseases; (6) adequate visual, auditory, and fine motor skills; and (7) not having any disability that limited independent walking. From the enrolled sample, 110 participants completed all assessment sessions.

### 2.3. Data Collection

**Figure 1:**
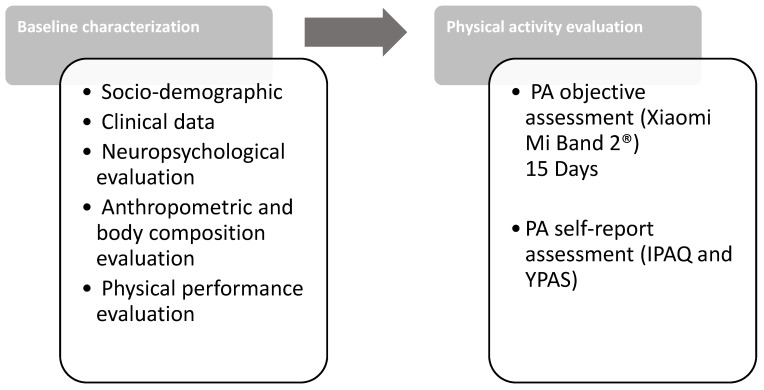
Flow diagram of the assessments.

The study had a cross-sectional study design. A baseline characterization was performed encompassing: (1) a structured questionnaire-based interview to assess socio-demographic and clinical information; (2) a neuropsychological evaluation to characterize depressive mood (Geriatric Depression Scale, GDS) [22] and global cognitive profiles (Mini-Mental State Examination, MMSE) [23]; (3) an anthropometric and body composition evaluation; and (4) physical performance evaluation (Figure 1). Lastly, self-reported PA was assessed with the International Physical Activity Questionnaire (IPAQ), long version [24], and The Yale Physical Activity Survey for older adults (YPAS) [25] through personal interviews. The Xiaomi Mi Band 2^®^ was used to objectively assess PA levels.

### 2.4. Neuropsychological Evaluation

The MMSE is the most used instrument for screening cognitive impairment. The Portuguese transcultural adaptation, standardization, and validity, were carried out by Guerreiro et al. (1994) [23]. Cognitive performance was classified according to the MMSE Portuguese version, the cut-off scores for “cognitive impairment” are as follows: individuals with no education, <15 points; 1 to 11 years of school completed, <22 points; and >11 years of school completed, <27 points [26]. The GDS was designed for rating depression in the elderly by Yesavage et al. (1983) [27]. GDS appears to be a valid and reliable test for use in depression screening in Portuguese elders Scores range from 0 to 30, with values >11 indicating the presence of depressive symptomatology [28].

### 2.5. Anthropometric Characterization

The anthropometric measures included weight (Tanita BF 350 Body Composition Analyzer), height (Seca 217 Stadiometer), and waist (WC) and hip circumferences (HP) (ergonomic measuring tape). Body mass index (BMI) was calculated as weight (kg)/height (m^2^). BMI was categorized as underweight, normal, overweight, and obese (respectively, BMI: 0–18.5, 18.6–24.9, 25.0–29.9, and 30.0+). The waist-to-hip ratio (WHR) was obtained by dividing the WC (cm) by HP (cm) circumference. WHR above 0.90 for males and above 0.85 for females was categorized as a substantially increased risk of metabolic complications [29].

### 2.6. Body Composition

Body composition was estimated by bioelectrical impedance analysis using a Maltron BioScan 920-II Multi-frequency Analyzer. The measurements were conducted according to manufacturer instructions. The cut-off values for body fat (FAT) percentage are ≤42% for women and ≤30% for men [30].

### 2.7. Physical Performance

Measurements of physical performance included gait speed (6-m walking test) and handgrip strength (Jamar Hand Dynamometer). Slow gait speed was classified as walking less than 1 m/s, which is the predictive value that identifies persons at high risk of health-related outcomes in well-functioning older people [31]. For handgrip strength the normative data ranging from 22.5 to 25.6 kg for women and 36.2 to 41.7 kg for men [32]. Functional status was assessed using the Lawton Instrumental Activities of Daily Living (IADL) scale to assess independent living skills [33], Katz Activities of Daily Living (ADL) scale to assess six primary and psychosocial functions (bathing, dressing, going to the toilet, transferring, feeding, and continence) [34], and Tinetti-test to assess the gait and balance [35].

### 2.8. International Physical Activity Questionnaire (IPAQ)

The IPAQ is the most widely used tool to collect self-reported PA. It was developed as an instrument for standardizing measures of the health-related PA behaviors of the population in multiple countries and different sociocultural contexts. Moreover, the validity and reliability of the IPAQ has been established in a 12-country evaluation, including Portugal [24]. The long-form of IPAQ comprises 27 items measuring the frequency and duration of PA performed in four domains, including occupational, transportation, household/gardening, and leisure-time activities, as well as sedentary activities, in the last seven days. The questionnaire was designed to provide separate domain-specific scores for walking, moderate-intensity, and vigorous-intensity activity, within each of the domains. The total score is estimated by summing all domains and intensity-specific PA minutes weighted by their metabolic equivalents (METs) and expressed in MET-minutes per week. According to the IPAQ scoring protocol, vigorous-intensity PA is defined as 8 METs, moderate-intensity activity 4 METs, and walking 3.3 METs [24,36]. The data collected in this study were analyzed following the IPAQ scoring protocol [36].

### 2.9. Yale Physical Activity Survey (YPAS)

The YPAS is an interviewer-administered questionnaire developed to assess PA in epidemiologic studies of community-dwelling older adults. The YPAS-PT version demonstrates acceptable validity and reliability to measure PA levels in the Portuguese older adults [25]. The questionnaire is divided into two sections allowing the estimation of PA in a typical week in the last month before evaluation. The first section comprises a detailed checklist about the type, duration, and intensity of 25 activities in five domains: household, yard work, caregiving, exercise, and recreational activities. The weekly PA energy expenditure (EE) is calculated by multiplying the total time spent in each activity by an intensity code (kcal.min^−1^) and then summed across all activities to create a weekly value (kcal.wk^−1^). The second section contains questions that estimate the frequency and duration in five distinct PA dimensions: vigorous activity, leisurely walking, moving, standing, and sitting. The index for each dimension is calculated by multiplying the frequency score by duration score and multiplying again by a weighting factor. A summary index of activity is the sum of the five individual indices. The weights are based on the intensity of the activity dimension [25,37,38].

### 2.10. Xiaomi Mi Band 2^®^

Participants were instructed to wear the wrist accelerometer continuously for fifteen consecutive days while performing their normal daily activities. The Master for Mi Band app was used to export data (steps and heart rate) in SQLite format then converted in CSV format.

Xiaomi Mi Band 2^®^ (15.7 mm × 10.5 mm × 40.3 mm and weighs 7.0 g) was selected among several commercially available wearable activity trackers because it has an estimated battery life of almost 30 days, is ergonomic, accessible, easy to operate, and offers a good price-quality ratio. The system combines sensors that allow the objective assessment of daily free-living PA, sleep, and heart rate [39,40,41,42]. Specifically, it has a triaxial accelerometer to detect movements, photoplethysmography sensor to monitor blood-volume changes, and combines a proximity sensor with actigraphy to detect sleep [40]. The algorithms used by the system are reliable to measure steps, intensity, energy expenditure, and distance traveled [39,40,41,42]. Moreover, Xiaomi Mi Band 2^®^ was tested for accuracy, precision, and validity. El-Amrawy has demonstrated that its high measurement accuracy (96.56%) and the relatively low variation coefficient (CV = 5.81) [41]. Furthermore, other authors also indicated a good accuracy and precision for this system [41,42].

### 2.11. Data Reduction

Data reduction and cleaning were performed using Microsoft Excel. From the 15 days of wear time, seven consecutive valid days were collected. The first day of recording was not included in the analysis. Data were included in the analysis if the participants had a minimum of 5 valid days, including at least one weekend day [12,16]. A valid day was defined as at least 10 h of records per day, and non-wear time as more than 30 consecutive minutes with zero activity [43,44]. The following PA intensity-specific cut-points were applied to the raw data: sedentary 0–19 steps/min; light intensity 60–99 steps/min, moderate activity 100–119 steps/min, and vigorous-intensity ≥120 steps/min [45,46]. Additionally, the older adults were classified using the following graduated step index: sedentary <5000 steps/day; low active 5000–7499 steps/day; somewhat active 7500–9999 steps/day active ≥10,000–12,499 steps/day; and highly active ≥12,500 steps/day [47,48].

### 2.12. Statistical Analysis

Data were analyzed using IBM SPSS Statistics (version 26). Descriptive statistics for continuous data are presented as mean ± standard deviation (SD). Categorical data are presented as absolute values and percentages. Normal data distribution was examined using the Shapiro–Wilk test, skewness, kurtosis, and histograms. Absolute values for skewness above 2.0 and kurtosis above 4.0 were considered as reference values for determining normality. Shapiro–Wilk test was used to validate the normality of all variables. A *p*-value below 0.05 indicates that the data significantly deviate from a normal distribution [49].

Gender differences on PA levels, anthropometric, body composition, and physical performance measures were assessed using independent sample t-test or Mann–Whitney U, depending on the normality of the variables. A *p*-value of less than 0.05 was considered statistically significant.

One sample T-test for the differences between accelerometer–measured and self-reported PA was used to determine if there is an agreement between the measurements. Furthermore, visual inspection of the Bland–Altman plot was used to explore the strength of agreement between the methods [50] and linear regression to determine if there is a proportional bias. Variables used for the Bland–Altman analysis were sedentary time, moderate, and vigorous PA, as well as total PA, according to IPAQ, YPAS, and Xiaomi Mi Band 2^®^.

Pearson or Spearman correlations were used to assess associations between accelerometer–measured PA, self-reported methods, and associations with health outcomes. The correlation coefficient was analyzed considering the Rule of Thumb [51].

Lastly, two-step cluster analysis was used to identify similar patterns of PA and sedentary behavior and association with health outcomes. Moderate PA, sedentary behavior, and meeting PA recommendations were clustering variables. Additionally, GDS, speed, BMI, and free fat mass (FFM) were included as evaluation fields. To establish how clusters that are statistically significant differed from one another, variables were examined using the Kruskal-Wallis H test.

## 3. Results

### 3.1. Study Participants

Table 1 summarizes the participants’ demographic, depressive mood, global cognitive, anthropometric, body composition, and physical performance characteristics. The mean age was 68.4 (SD ± 3.1), 60% were female, and the mean number of years of formal education was 8.0 (SD ± 5.4) (number of school years).

According to cut-offs of the Portuguese version of MMSE all participants were classified as without cognitive impairment (27.0 ± 2.0). The GDS results indicated no presence of depressive symptoms (6.1 ± 4.6). The average BMI was 28.5 (SD ± 4.3), which is categorized as overweight, and the WHR ratio was 0.95 (SD ± 0.07), meaning that this sample of older adults had a substantially increased risk of metabolic complication. Results did not show statistical differences between genders for BMI or WHR. Women had significantly higher fat mass (%) 38.3 (SD ± 6.3) compared to men 27.8 (SD ± 5.2). The values are categorized within optimal values for both genders. The gait speed and handgrip strength were in the range of reference values, indicating good physical performance. The functional status evaluation confirmed the independence in activities of daily living and performance of instrumental activities of daily living.

The descriptive data from IPAQ, YPAS, and Xiaomi Mi Band 2^®^ are presented in Table 2, Table 3 and Table 4, respectively. Overall, the activities self-reported were predominantly walking, household, leisure, and gardening activities. Furthermore, the results have shown a significant difference in PA measured by IPAQ between gender for domestic activities, sitting time, moderate activities, and total PA (Table 2), with higher values for women. Similarly, results from PA measured by YPAS also showed significant differences in domestic activities and total PA (Table 3).

Xiaomi Mi Band 2^®^-measured PA have shown that the participants spent more time in light or moderate activities (275.4 ± 392.7, 120.5 ± 127.9 min.wk^−1^, respectively), compared to vigorous activities (62.4 ± 113.5 min.wk^−1^) (Table 4). On the other hand, no significant differences were observed between men and women in total minutes per day spent in all PA intensities, as measured by the Xiaomi Mi Band 2^®^.

### 3.2. Correlation Between Self-Reported and Accelerometer-Based Estimates of PA

Correlations between Xiaomi Mi Band 2^®^ and PA measured by IPAQ and are shown in Table 5. A significant low positive correlation was found for sedentary time, vigorous-intensity, and total PA. For moderate-intensity, the results indicate a negligible correlation between accelerometer and self-reported PA. Concerning the correlations between Xiaomi Mi Band 2^®^ and PA measured by YPAS, a significant moderate positive correlation (r = 0.42, *p* = 0.001) for vigorous-intensity was found (Table 5).

### 3.3. Agreement Between Self-Reported and Accelerometer-Based Estimates of PA

Analyses comparing the concordance of values between the Xiaomi Mi Band 2^®^ and IPAQ showed that older adults reported both less sedentary time and less vigorous-intensity time compared to the accelerometer measured data. Moreover, there was a significant difference between the two methods for all intensities, except for vigorous-intensity time; meaning that the two methods only agree on the estimation of vigorous-intensity time (*p* = 0.60) (Table 6). Regarding the concordance of absolute values between the Xiaomi Mi Band 2^®^ and YPAS, we observed higher self-reported PA for all intensities tested. Additionally, we observed that the two methods are significantly different from one another (*p* = 0.001) (Table 6).

**Figure 2:**
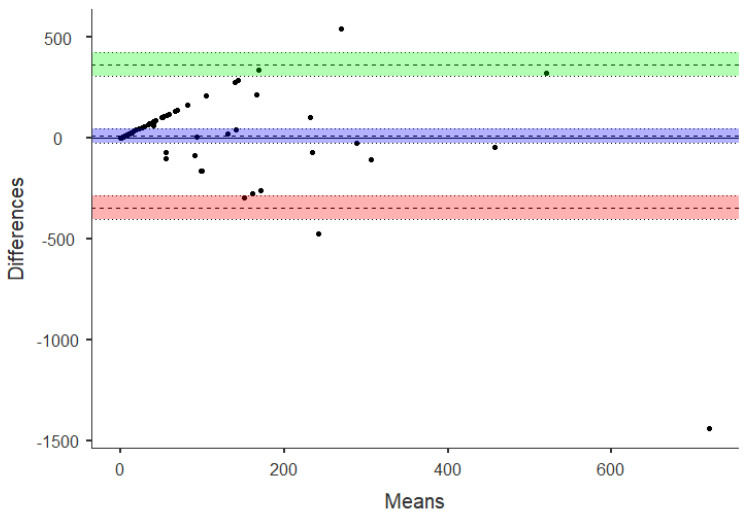
Bland–Altman plot for vigorous PA assessed by IPAQ and Xiaomi Mi Band 2^®^. The mean difference (systematic error) and 95% limits of agreement (mean ± 1.96 SD) and confidence interval shadings are displayed in the figure.

**Figure 3:**
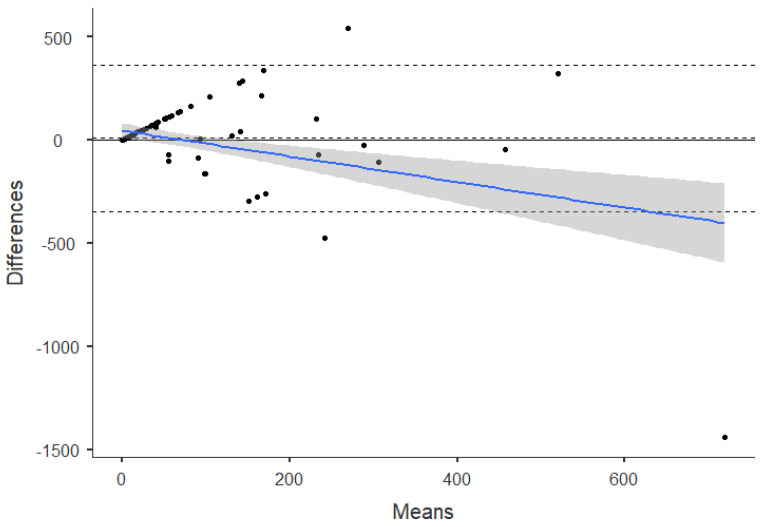
Bland–Altman plot for vigorous PA assessed by IPAQ and Xiaomi Mi Band 2^®^. The mean difference (systematic error) and 95% limits of agreement (mean ± 1.96 SD), proportional bias line, and proportional bias line confidence intervals are displayed in the figure.

Since a possible agreement between vigorous-intensity time obtained from the Xiaomi Mi Band 2^®^ and IPAQ was found, the Bland–Altman plot was used to explore the strength of this agreement. Figure 2 and Figure 3 show the differences between Xiaomi Mi Band 2^®^–measured PA and IPAQ plotted against the mean of the accelerometer and IPAQ for vigorous PA minutes per week. Overall, the mean difference was 9.1 ± 180.9 min.wk^−1^, and the limits of agreement presented a higher variation, ranging from –345.4 to 363.6 min.wk^−1^. The mean of the data lies considerably above 0, indicating that the Xiaomi Mi Band 2^®^ estimates are consistently greater than IPAQ estimates. Moreover, the cluster of points showed a trend or error proportional to the size of the measure.

Finally, the results from the regression analysis of the differences versus mean values confirms a significant systematic bias in the methods agreement (*p* < 0.000).

### 3.4. Association Between PA Estimates and Health Outcomes

The association between health outcomes variables with Xiaomi Mi Band 2^®^–measured and self-reported PA are presented in Table 7. IPAQ estimates show a moderate positive correlation with muscle mass and FFM, and a weak positive correlation with weight, WHR, and speed. Similarly, YPAS estimates also show a moderate positive correlation with muscle mass and FFM, and a weak positive correlation with weight. Concerning Xiaomi Mi Band 2^®^–measured PA, results reveal a weak positive correlation between a Xiaomi Mi Band 2^®^–measured PA and GDS, gait, and speed.

### 3.5. Physical Activity Profile

Forty-eight percent of the participants (25.5% males and 22.7% females) were found to meet the guidelines of a minimum of 8000 steeps/day (Table 8). In this sample of older adults, 45.5% had a sedentary behavior (17.3% sedentary, 28.2% low active) and 54.7% active behavior (25.5% somewhat active, 14.6% active, and 14.6% highly active).

### 3.6. Cluster Analysis of Physical Activity and Sedentary Behavior

**Figure 4:**
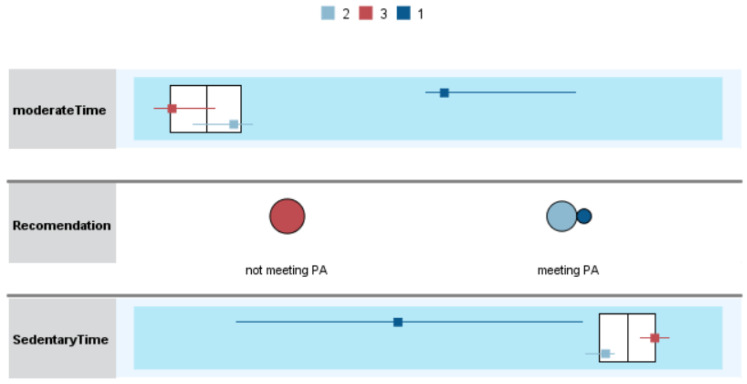
Cluster comparison.

The analysis revealed three distinct clusters with a silhouette measure of cohesion and separation of 0.8. Kruskal–Wallis H test confirmed that individual clusters differed significantly from each other. The different clusters showed the following consistent rank order of PA levels: C1 > C2 > C3. Cluster 1 displayed higher moderate PA levels, lower sedentary behavior, and meeting PA recommendations. In cluster 2, participants also met PA recommendations but had less moderate PA levels, and higher sedentary behavior, compared to cluster 1. On other hand, cluster 3 displayed low moderate PA levels, higher sedentary behavior, and participants did not meet the PA recommendations (Figure 4).

Regarding the participant’s characteristics within the clusters, GDS, speed, BMI, and FFM were significantly different, with better health-related outcomes in clusters that comprised the participants that meeting the PA recommendations (C1 and C2) (Table 9).

## 4. Discussion

There is consistent evidence that PA can positively impact the aging process, for instance in physical and neuropsychological dimensions [52]. However, it is unclear the quantification of the strength and nature of the relationship between PA and health outcomes. Consequently, accurate assessment of PA is crucial for quality research across the public health and exercise science fields, with repercussions in the clinic. Here, in a cohort of older adults, the main objective was to compare accelerometer-based estimates of PA (using a Xiaomi Mi Band 2^®^) with commonly used self-report questionnaires. Furthermore, the associations between PA and health outcomes were explored and PA profiles analyzed.

Overall, results show a poor to moderate correlation between self-reported and accelerometer-based estimates of PA and sedentary behavior. Briefly, and in line with previous findings [14,21,44,53], low positive correlation coefficients were observed for IPAQ estimates (sedentary behavior, vigorous PA, and total PA) and moderate positive correlation for YPAS estimates of vigorous PA (r = 0.42, *p* < 0.001). The results also demonstrate a large variation between self-reported and Xiaomi Mi Band 2^®^-measured PA and sedentary time. As expected, the highest difference between objective and subjective methods was found for the sedentary time (7357.8 ± 940.9 for IPAQ and −885.0 ± 869.0 YPAS), which follows a systematic review from Prince et al. (2020) indicating that self-report measures generally underestimate sedentary time when compared to device measures [17]. Surprisingly, we observed the lowest variation for vigorous PA. This result was unexpected since other authors have observed that the difference between the self-reported and objective measures increases with higher PA intensity levels [18]. Furthermore, a higher Xiaomi Mi Band 2^®^-vigorous-intensity PA was noted when comparing with self-reported vigorous-intensity PA, whereas other studies have found higher values of self-reported vigorous-intensity PA [17,18,19].

Since the correlation coefficient only evaluates the relationship between the variables and not the differences, it is not the best method for assessing comparability [54]. Alternatively, the Bland–Altman plot quantifies the agreement between objective versus subjective methods. Overall, the results confirmed the lack of agreement between self-reported and accelerometer-based estimates of PA. Specifically, Xiaomi Mi Band 2^®^–measured PA minutes per day was plotted against the mean of the self-report and accelerometer for vigorous PA for IPAQ estimates. The mean difference was 9.1 ± 180.9 min.wk^−1^ with the limits of agreement ranging from –345.4 to 363.6 min.wk^−1^. Results indicate that the Xiaomi Mi Band 2^®^ estimates of vigorous PA are consistently greater than IPAQ estimates. Additionally, the Bland–Altman plot for vigorous-intensity PA (IPAQ) provides evidence of proportional bias. These findings are consistent with previous studies and highlight the fact that measuring PA levels in the same person with these instruments leads to significant variations in magnitude and agreement [14,21,53].

Altogether, the present findings confirm that the general agreement between self-reported and accelerometer-measured PA is poor, and the correlation coefficients lower than what is recommended [18]. The lack of correlation and agreement between methods may be explained by the fact that the instruments do not measure the same constructs [14,44]. While the accelerometer measures the motion through acceleration, and uses laboratory-derived intensity thresholds to determine how much time was spent at different intensities of movement (sedentary, light, moderate, and vigorous), the questionnaires measure the time spent in specific domains and intensities [14,44]. Furthermore, questionnaires use information based on self-report minutes of activity performed according to intensity, with a risk of misinterpretation of intensity associated with difficulties in remembering the frequency and duration of several activities especially in older adults [44].

Regarding the study population, the assessment results indicated no limitations in cognitive function, physical function, and functionality, with all individuals being fully independent in daily living activities. The sample was categorized as overweight according to the BMI and the WHR ratio criteria, which are associated with metabolic risk factors. Other studies in the Portuguese population have shown that more than 20% of the adults are obese with an obesity prevalence higher in women [55]. As expected, the body composition measures showed that women had significantly higher fat mass when compared to men and lower fat-free mass and muscle mass, with the values categorized within optimal values for both genders. In a recent study, Pereira et al. assessed body composition in a sample of Portuguese centenarians and observed mean values of FAT and FFM inferior to our study [56]. Indeed, aging is associated with changes in body composition mainly expressed in changes in body fat distribution and loss of muscle mass [56,57]. Therefore, the assessment of body composition changes in the aging process could provide insights about the optimal weight for health status and physical performance [56,58]. The combination of low muscle mass and poor muscle function, i.e., sarcopenia, is a geriatric condition associated with adverse effects on function, quality of life, and survival [57]. Moreover, high body fat has been associated with poorer physical performance in older adults, and fat accumulation within the skeletal muscle is associated with muscle weakness and poor function [58]. Remarkably, we observed that gender differences were found for most of the evaluated body composition parameters. A similar pattern was obtained in the aforementioned study in Portuguese centenarians [56]. Lastly, we observed a higher prevalence of obesity using anthropometric measures comparing to bioimpedance analysis, these results are in line with a previous study [56].

Sedentary behavior has been described as an independent risk factor for poor health and mortality and is associated with obesity, muscle weakness, and mobility disability in older adults [59]. Concerning our study sample, the majority spent most of their daily activity in sedentary behavior. On average they spent 9088.9 min/week (SD ± 735.7) in accelerometer-determined sedentary behavior, with no significant differences between genders. However, self-reported sedentary behavior (IPAQ estimates) was 247.4 ± 127.3 with men reporting significantly higher levels. Therefore, study findings indicate that the participants assume that they are substantially less sedentary than they really are. A similar pattern was found in other studies [17,60]. The reported activities were predominantly walking, household, leisure, and gardening [61]. Also, a recent systematic review found that walking is the most frequently performed activity [18]. Moreover, results show a significant difference in PA between genders on domestic activities, sitting time, moderate activities, and total PA (IPAQ estimates), and in domestic activities and total PA (YPAS estimates), with higher values for women. Regarding levels of PA reported (IPAQ estimates) a significant difference for all intensities, except for vigorous-intensity, is noted. Therefore, the gender differences seem to influence these estimates only for self-reported PA. Concerning Xiaomi Mi Band 2^®^-measured PA, in general, participants spent more time in light compared to moderate or vigorous activities, as expected. No differences between gender in total minutes per day spent in all intensities for Xiaomi Mi Band 2^®^-measured PA were found. Approximately 45% of individuals have a sedentary behavior and 48% are categorized as having met PA recommendations. According to the American College of Sports Medicine (ACSM), the older adult’s recommendation is 150 min of PA with moderate-intensity or 75 min of vigorous-intensity, or an equivalent combination of moderate and vigorous PA per week to promote health [62]. Promoting PA in older adults is crucial to avoid the deterioration of physiological functions, body composition, physical function, and loss of mobility.

Concerning the association between health outcome variables, the results reveal a weak positive correlation between a Xiaomi Mi Band 2^®^–measured PA and GDS and gait speed. For IPAQ self-reported PA we found a moderate positive correlation with muscle mass and FFM, and a weak positive correlation with weight, WHR, GDS, and gait speed. Equally, YPAS self-reported PA shows a moderate positive correlation with muscle mass and FFM, and a weak positive correlation with weight. This finding was consistent with previous studies showing that self-reported and objectively measured PA associate differently with health outcomes. Future research is needed to better understand why different types of measures associate differently with health outcomes [17]. Regarding the clusters analysis, we observed that the clusters C1 and C2, in which the participants meet the PA recommendations and with higher levels of PA, show better results in the evaluated health outcomes (GDS, speed, BMI, and FFM), as expected.

The strengths of this study are that the self-reported and the accelerometer data represent the same periods and provide new information about several physical functions, body composition variables, and PA levels in older adults, which is important for the knowledge of healthy aging strategies. Nonetheless, the small sample size and cross-sectional nature of the work limit (causality) inferences regarding the associations between PA and health outcomes. Moreover, the sample is not representative of the Portuguese older population, and a “healthy participant effect” must be considered; thus, findings cannot be widely generalizable. Concerning the limitations associated with accelerometry-based devices, it should be noted a low-sensitivity for sedentary behaviors, the limited ability for assessing low-intensity activities, and the lack of standard or generally acceptable placements for them [63]. Cleland et al. (2013) evaluated the optimal placement of accelerometers and found that the hip was the best single location to record data. The lower accuracy for data from the wrist may be due, in part, to arm movements unassociated with the measured activity. Furthermore, they observed that by combining data from accelerometers on the wrist and hip the accuracy increased [64]. Boerema et al. also mentioned the influence of the placement, type of activity, and their interaction-effect. The authors recommended that to increase reliability and to reduce the variability, the optimal placement should be on the most lateral position of a participant’s waist belt [65]. Another limitation to consider is the cut-off point for accelerometer-measured sedentary behavior, which includes activities such as sitting and standing. Thus, this could increase the difference between the two methods since questionnaires only asks about time spent sitting and not standing and the accelerometer included standing time [18]. Finally, the choice of accelerometer cut points has a large influence on the comparison of the absolute results.

In summary, this study found large differences, a low correlation, and absence of agreement, between self-reported and accelerometer-based estimates of PA, in a cohort of older adults, but the comparison between the two methods provides valuable information about habitual PA levels in senior populations.

## Figures and Tables

**Table 1:** General characteristics of the study participants.

Characteristics	Total Sample *n* = 110	Male *n* = 50	Female *n* = 60	*p*-Value
**Gender, *n* (%)**		50 (45.5)	60 (54.5)	
**Age, years (mean ± SD)**	68.4 ± 3.1	69.4 ± 2,9	67.5 ± 3.0	<0.000
**Education, years (mean ± SD)**	8.0 ± 5.4	9.4 ± 6.0	6.8 ± 4.6	0.008
**MMSE, total score (mean ± SD)**	27.0 ± 2.0	27.1 ± 1.8	26.8 ± 2.1	0.57
**GDS, total score (mean ± SD)**	6.1 ± 4.6	5.3 ± 3.9	6.7 ± 5.0	0.25
**Anthropometric (mean ± SD)**				
**Weight, Kg**	73.6 ± 13.0	79.0 ± 11.8	69.2 ± 12.4	<0.000
**Height, m^2^**	159.7 ± 8.36	166.3 ± 6.4	154.2 ± 5.3	<0.000
**BMI, kg/m^2^**	28.5 ± 4.3	27.8 ± 3.5	29.0 ± 4.9	0.65
**WC, cm**	99.4 ± 9.8	100.2 ± 9.7	98.7 ± 9.7	0.43
**HP, cm**	104.8 ± 9.03	103.3 ± 6.9	106.0 ± 10.4	0.29
**WHR**	0.95 ± 0.07	0.97 ± 0.08	0.93 ± 0.06	0.013
**Body composition (mean ± SD)**				
**FAT, %**	33.5 ± 7.8	27.8 ± 5.2	38.3 ± 6.3	<0.000
**FAT, Kg**	24.7 ± 8.6	21.5 ± 6.5	27.3 ± 9.2	<0.000
**FFM, %**	66.5 ± 7.8	72.2 ± 5.2	61.7 ± 6.3	<0.000
**FFM, Kg**	48.2 ± 9.2	54.8 ± 7.6	42.7 ± 6.4	<0.000
**Mineral, Kg**	4.0 ± 0.5	4.2 ± 0.5	3.8 ± 0.4	<0.000
**Muscle, Kg**	23.7 ± 4.2	26.5 ± 3.6	21.3 ± 3.0	<0.000
**BCM, Kg**	27.5 ± 4.9	30.9 ± 4.2	24.7 ± 3.4	<0.000
**Malnutrition Index**	0.66 ± 0.05	0.69 ± 0.05	0.64 ± 0.04	<0.000
**Physical performance (mean ± SD)**				
**ADL score**	5.7 ± 0.5	5.9 ± 0.4	5.5 ± 0.6	0.002
**IADL score**	8.0 ± 0.1	8.0 ± 0.1	7.9 ± 0.2	0.67
**Balance score**	15.6 ± 0.7	15.5 ± 0.7	15.6 ± 0.8	0.28
**Gait score**	12.0 ± 0.2	12.0 ± 0.1	12.0 ± 0.1	0.1
**Max Right Grip, Kg**	31.0 ± 10.1	39.4 ± 8.0	24.0 ± 4.8	<0.000
**Max Left Grip, Kg**	29.6 ± 9.6	37.2 ± 8.6	23. ± 4.4	<0.000
**Speed, m/sec**	1.7 ± 0.3	1.8 ± 0.3	1.6 ± 0.3	<0.000

Abbreviations: MMSE, Mini Mental State Examination; GDS, Geriatric Depression Scale; BMI, Body mass index; WC, waist circumference; HP, hip circumference; WHR, waist-to-hip ratio; FAT, Fat Mass; FFM, Free Fat Mass; Muscle, Muscle mass; Mineral, Mineral body density; BCM, Body Cell Mass; ADL, Activities of Daily Living; IADL, Instrumental Activities of Daily Living.

**Table 2:** Descriptive PA data from International Physical Activity Questionnaire.

Variable	Total Sample *n* = 110	Male *n* = 50	Female *n* = 60	*p*-Value
Total Work (min.d^−1^)	218.4 ± 1272.8	209.6 ± 1201.7	225.7 ±1339.2	0.53
Total Transport (min.d^−1^)	698.5 ± 1013.0	903.5 ± 1107.2	527.7 ± 901.5	0.030
Total Domestic (min.d^−1^)	3789.9 ± 3948.5	1976.6 ± 3074.9	5301.0 ± 3980.2	<0.000
Total Leisure (min.d^−1^)	1248.6 ± 1577.4	1212.1 ± 1343.5	1279.0 ± 1759.4	0.99
**Total Average Sitting (min.d^−1^)**	247.4 ± 127.3	288.4 ± 149.0	213.4 ± 94.4	0.008
Total Walking (min.d^−1^)	1262.9 ± 1752.6	1313.4 ± 1292.3	1220.7 ± 2069.7	0.084
Total Vigorous (MET.min.d^−1^)	333.3 ± 828.4	432.7 ± 921.4	250.3 ± 739.8	0.22
Total Moderate (MET.min.d^−1^)	4359.3 ± 3884.9	2555.6 ± 3176.1	5862.3± 3801.4	<0.000
**Total MET (MET.min.d^−1^)**	5955.4 ± 3957.1	4301.8 ± 3380.0	7333.4 ± 3897.6	<0.000

Abbreviations: PA, physical activity; min.d^−1^; minutes per day, MET.min.d^−1^, metabolic equivalent of task minutes per day.

**Table 3:** Descriptive PA data from Yale Physical Activity Survey.

Variable	Total Sample *n* = 110	Male *n* = 50	Female *n* = 60	*p*-Value
**Household Total Time (min.wk^−1^)**	1022.4 ± 805.5	476.6 ± 520.4	1477.2 ± 714.8	<0.000
**Household Total EE (kcal.wk^−1^)**	3097.9 ± 2406.4	1542.7 ± 1566.8	4394.0 ± 2214.0	<0.000
**Yard work Total Time (min.wk^−1^)**	209.7 ± 514.4	137.6 ± 253.4	269.8 ± 653.8	0.71
**Yard work Total EE (kcal.wk^−1^)**	965.6 ± 2338.9	637.6 ± 1172.4	1238.9 ± 2966.1	0.71
**Caregiving Total Time (min.wk^−1^)**	124.4 ± 396.6	118.8 ± 279.5	129.0 ± 432.9	0.54
**Caregiving Total EE (kcal.wk^−1^)**	578.1 ± 1645.8	566.1 ± 1382.3	588.0 ± 1848.6	0.54
Exercise Total Time (min.wk^−1^)	218.2 ± 230.0	216.9 ± 234.2	219.3 ± 228.4	0.88
Exercise Total EE (kcal.wk^−1^)	1268.1 ± 1510.8	1217.7 ± 1327.4	1310.0 ± 1658.2	0.90
**Leisure Total Time (min.wk^−1^)**	117.9 ± 252.0	136.3 ± 299.1	102.6 ± 206.1	0.99
Leisure Total EE (kcal.wk^−1^)	388.8 ± 859.9	512.8 ± 1144.6	285.5 ± 504.1	0.87
**Total PA Time (min.wk^−1^)**	1692.6 ± 1107.0	1086.2 ± 821.4	2197.9 ± 1064.0	<0.000
Total EE (kcal.wk^−1^)	6298.4 ± 4152.3	4476.8 ± 3265.3	7816.4 ± 4224.1	<0.000
**Vigorous activity index (units.month^−1^)**	4.7 ± 13.1	7.5 ± 17.1	2.3 ± 7.9	0.16
**Leisurely walking index (units.month^−1^)**	14.6 ± 14.8	15.7 ± 14.3	13.7 ± 15.3	0.33
Moving index (h.d^−1^)	12.1 ± 3.7	11.0 ± 3.6	13.0 ± 3.5	0.003
Standing index (h.d^−1^)	1.0 ± 1.3	1.1 ± 1.4	1.0 ± 1.3	0.81
Sitting index (h.d^−1^)	1.8 ± 0.7	1.9 ± 0.7	1.6 ± 0.6	0.015
Stair climbing (units.d^−1^)	9.5 ± 12.5	9.9 ± 14.2	9.1 ± 11.0	0.78
Summary index (total units)	34.1 ± 20.4	37.2 ± 23.7	31.6 ± 16.9	0.42
Seasonal Adjust	1.2 ± 2.0	1.4 ± 3.0	1.00 ± 0.10	0.76

Abbreviations: PA, physical activity; min.wk^−1^, minutes per week; EE, energy expenditure; kcal.wk^−1^, kilocalorie per week; h.d^−1^, hours per day.

**Table 4:** Descriptive PA data from Xiaomi Mi Band 2^®^.

Variable	Total Sample *n* = 110	Male *n* = 50	Female *n* = 60	*p*-Value
Sedentary time (min.wk^−1^)	9088.9 ± 735.7	9084.6 ± 639.0	9092.5 ± 813.0	0.33
Light PA (min.wk^−1^)	275.4 ± 392.7	278.4 ± 316.3	272.9 ± 449.2	0.045
Moderate PA (min.wk^−1^)	120.5 ± 127.9	116.5 ± 91.4	123.8 ± 152.5	0.40
Vigorous PA (min.wk^−1^)	62.4 ± 113.5	63.5 ± 132.6	61.4 ± 96.0	0.14
**Total PA Time (min.wk^−1^)**	991.1 ± 735.7	995.4 ± 634.0	987.5 ± 813.0	0.33

Abbreviations: PA, physical activity; min.wk^−1^.

**Table 5:** Spearman correlations between Xiaomi Mi Band 2^®^ and self-reported PA (IPAQ and YPAS).

Variable	r	*p*-Value
**International Physical Activity Questionnaire**		
Sedentary time	0.38	<0.000
Moderate time	0.04	0.70
Vigorous time	0.30	0.002
Total PA time	0.27	0.004
**Yale Physical Activity Survey**		
Moderate time	0.003	0.97
Vigorous time	0.42	<0.000
Total PA time	0.23	0.017

**Table 6:** Mean difference in minutes per week between Xiaomi Mi Band 2^®^ and self-reported PA (IPAQ and YPAS).

Variable	Mean Difference ± SD	*p*-Value
**International Physical Activity Questionnaire**		
Sedentary time	7357.8 ± 940.9	<0.000
Moderate time	−1191.1 ± 1176.7	<0.000
Vigorous time	9.1 ± 180.9	0.60
Total PA time	−767.9 ± 1234.4	<0.000
**Yale Physical Activity Survey**		
Moderate time	−885.0 ± 869.0	<0.000
Vigorous time	−190.9 ± 247.2	<0.000
Total PA time	−701.5 ± 1076.6	<0.000

Note: Positive mean difference value: Xiaomi Mi Band 2^®^ PA is higher than self-reported PA. Negative mean differencevalue: Xiaomi Mi Band 2^®^ PA is lower than self-reported PA.

**Table 7:** Associations between Xiaomi Mi Band 2^®^–measured PA and the mean difference in minutes per week between Xiaomi Mi Band 2^®^ and self-reported PA (IPAQ and YPAS) with health outcomes.

	Weight	WHR	BMI	FFM	Muscle	Education	GDS	Gait	Speed
**International Physical Activity Questionnaire**									
r	−0.30	−0.24	−0.037	−0.39	−0.40	−0.092	−0.084	0.13	−0.22
*p*-value	0.001	0.014	0.07	<0.000	<.000	0.339	0.382	0.182	0.020
**Yale Physical Activity Survey**									
r	−0.30	−0.20	−0.007	−0.48	−0.48	−0.26	0.040	0.039	−0.19
*p*-value	0.001	0.037	0.94	<0.000	<0.000	0.006	0.68	0.68	0.050
**Xiaomi Mi Band 2^®^**									
r	−0.13	−0.071	−0.14	−0.010	−0.008	−0.076	−0.23	0.22	0.28
*p*-value	0.19	0.47	0.14	0.92	0.94	0.43	0.017	0.022	0.003

Abbreviations: WHR, waist-to-hip ratio; BMI, Body mass index; FFM, Free Fat Mass; GDS, Geriatric Depression Scale.

**Table 8:** Classification of older individuals according to the current public health PA recommendations.

Characteristics	Total Sample *n* = 110	Male *n* = 50	Female *n* = 60
**Classification, *n* (%)**			
Sedentary	19 (17.3)	7 (6.4)	12 (10.9)
Low active	31 (28.2)	13 (11.8)	18 (16.4)
Somewhat active	28 (25.5)	15 (13.6)	13 (11.8)
Active	16 (14.6)	8 (7.3)	8 (7.3)
Highly active	16 (14.6)	7 (6.4)	9 (8.2)
**PA recommendation, *n* (%)**			
Meeting PA	53 (48.2)	28 (25.5)	25 (22.7)
Not meeting PA	57 (51.8)	22 (20.0)	35 (31.8)

**Table 9:** Descriptive statistic for each cluster.

Characteristics	C1 *n* = 10	C2 *n* = 43	C3 *n* = 57	*p*-Value
PA recommendation, *n* (%)				
Meeting PA	10 (9)	43 (39)	0	
Not Meeting PA	0	0	57 (52)	
Sedentary time (mean ± SD)	7451.9 ± 1507.5	8998.8 ± 232.0	9444.1 ± 186.7	<0.000
Light PA (mean ± SD)	1091.8 ± 966.7	269.8 ± 100.9	136.4 ± 59.6	<0.000
Moderate PA (mean ± SD)	455.5 ± 154.4	119.2 ± 56.8	62.7 ± 46.3	<0.000
Vigorous PA (mean ± SD)	226.2 ± 236.3	88.5 ± 102.9	13.9 ± 21.3	<0.000
Total PA (mean ± SD)	2628.1 ± 1507.5	1081.2 ± 232.0	635.9 ± 186.7	<0.000
Speed (mean ± SD)	1.8 ± 0.3	1.8 ± 0.3	1.6 ± 0.3	<0.000
GDS (mean ± SD)	5.1 ± 3.5	4.6 ± 3.8	7.3 ± 5.0	0.012
BMI (mean ± SD)	26.7 ± 1.8	27.9 ± 3.4	29.9 ± 4.8	0.020
FFM (mean ± SD)	67.8 ± 6.0	69.3 ± 7.4	64.1 ± 7.8	0.006

## Data Availability

Research data presented in this study are available on request from the corresponding author.
